# CSF-1 regulates the function of monocytes in Crohn’s disease patients in remission

**DOI:** 10.1038/s41598-017-00145-4

**Published:** 2017-03-07

**Authors:** Juan Camilo Nieto, Carlos Zamora, Elisabet Cantó, Esther Garcia-Planella, Jordi Gordillo, Maria Angels Ortiz, Cándido Juárez, Silvia Vidal

**Affiliations:** 1 Institut Recerca Hospital de la Santa Creu i Sant Pau, Barcelona, Spain; 2 Department of Gastroenterology, Hospital de la Santa Creu i Sant Pau, Barcelona, Spain; 3 Department of Immunology, Hospital de la Santa Creu i Sant Pau, Barcelona, Spain

## Abstract

During the flare-ups of Crohn’s disease (CD) patients, circulating leukocytes actively migrate toward the inflamed sites. During the remission, the lack of symptoms does not necessarily imply immunological remission. To decipher inflammatory mechanisms still operating during CD remission, we compared the expression of chemokine receptors on monocytes from CD and healthy donors (HD), and how these differences could modulate monocyte maturation and cytokine production. Flow cytometry analysis showed a higher expression of CCR5 on monocytes from CD patients than those from HD after 24 h. This CCR5 upregulation was associated with the spontaneous production of CSF-1 and IL-10. The higher expression of CCR5 on CD monocytes increased their migratory pattern in response to CCL5. Signaling through CCR5/CCL5 increased CD163 and HLA-DR expression and diminished TLR4-induced TNF-α and IL-6 secretion during monocyte differentiation. When we analyzed clinical parameters, patients treated with azathioprine had the highest CSF-1 levels and CCR5 expression. Our results suggest that monocytes from CD patients in remission produced high levels of CSF-1 that upregulate CCR5 expression. Consequently, monocytes differentiated in these conditions had a characteristic phenotype and lower production of inflammatory cytokines. The treatment with azathioprine could be responsible for this anti-inflammatory profile of monocytes.

## Introduction

Crohn’s disease (CD) is a disorder of the gastrointestinal mucosa, characterized by the infiltration of immune cells into the lamina propria. In recent studies, monocytes and monocyte-derived macrophages have appeared to be crucial players in the chronic inflammation of CD patients^1, 2^.

Peripheral blood monocytes are recruited at sites of inflammation toward chemokines, and they are the source of macrophages in inflamed intestinal mucosa^3, 4^. Monocytes express a wide panel of inflammatory chemokine receptors, including CCR2, CCR5, CXCR4, and CX3CR1^5^. During the maturation into tissue macrophages, monocytes are accompanied by a loss of CCR2 and an increased expression of CCR1 and CCR5^6, 7^. These chemokine receptor switches on the monocyte might indicate that the CCL2/CCR2 axis is involved in the initial recruitment, followed by a subsequent CCR5/CCL5-dependent migration into the tissues^8^. These receptors and their chemokines play a key role in the immune system’s defense, not only by directing the migration, but also by regulating the inflammatory profile of the monocytes. For example, CCL5 modulates the TLR4-induced production of TNF-α and IL-6 by human monocytes^9^, and CXCL12 production modulates monocyte differentiation to macrophages through CXCR4^10^. These unexpected functions suggest the influence of chemokines on the immunopathology of inflammatory bowel disease (IBD)^11, 12^. Several examples have demonstrated this influence *in vivo*: CCR2 knock-out (KO) and CCR5 KO mice were protected from dextran-sulfate sodium (DSS)-induced intestinal adhesions, mucosal ulcerations, and colonic inflammation as a consequence of the IL-10 increase and the IFN-γ decrease; CCL2/MCP-1 KO mice have a markedly reduced severity of colitis and reduced mortality rate^13^. In regard to patients, the mucosa CCL5 expression was higher in active CD patients than in healthy donors (HD), and it seems that CCL5 secretion could be regulated by steroid treatment^14–16^. The intestinal CCL2 expression, which was also higher in IBD patients than in HD, contributed to the chemoattraction of monocytes to the inflamed mucosa^17^. The expression of chemokine receptors was also different on leukocytes from CD patients. Non-classical monocytes from active CD patients expressed higher levels of CX3CR1 and low levels of CCR2 than HD. Inversely, the expression of CCR2 in classical monocytes from CD patients was higher than in these monocytes from HD^18, 19^. When monocytes from CD patients in remission were primed with lipopolysaccharide (LPS), they secreted less IL-10 and more CCL2 and CCL5. However, no differences in adhesion, phagocytosis, or the production of reactive oxygen were found between inactive CD patients and HD^20^. Although it has been demonstrated that blood monocytes from CD patients are recruited to the inflamed gut mucosa^21^, chemokine expression and the migratory pattern of CD monocytes from patients in remission have not been well characterized.

The major goal of current treatments in IBD is to increase the number and length of periods in clinical remission. However, lack of clinical symptoms in IBD patients does not necessarily imply biochemical, endoscopic, histologic and immunological remission. We hypothesize that the expression of chemokine receptors on blood monocytes from CD patients in remission is different from HD. This different profile of chemokine receptors could modulate not only their migratory pattern, but also the monocyte maturation and cytokine response. For this purpose, we assessed peripheral blood monocytes from CD patients in remission, their chemokine receptors expression (CCR2, CCR5, CXCR4 and CX3CR1), migratory capacity, the influence of chemokines during monocyte maturation and cytokine production, and their relationship with clinical parameters and the development of the disease.

## Results

### Expression of chemokine receptors on monocytes from CD patients in remission

To assess the expression of chemokine receptors, fresh and 24 h-whole blood (WB) cultured monocytes from CD patients and HD were stained with anti-CD14, anti-CD16 and anti-CCR2, CCR5, CXCR4, and CX3CR1 and were analyzed by flow cytometry (Supplementary Fig. S1). There were no differences in the expression of CCR2, CXCR4 and CX3CR1 on CD and HD fresh monocytes. However, the percentage of CCR5+ monocytes was slightly higher in CD than in HD (HD: 16.20 ± 1.20, CD: 21.90 ± 2.08; p < 0.05). After 24 h in WB culture, the percentage of CCR5+ monocytes was even higher in CD than in HD (HD: 49.53 ± 2.17, CD: 73.07 ± 2.70; p < 0.001) (Supplementary Fig. S1). No significant differences were observed in the CCR2, CXCR4, and CX3CR1 expression of 24 h-cultured HD and CD monocytes.

**Figure 1:**
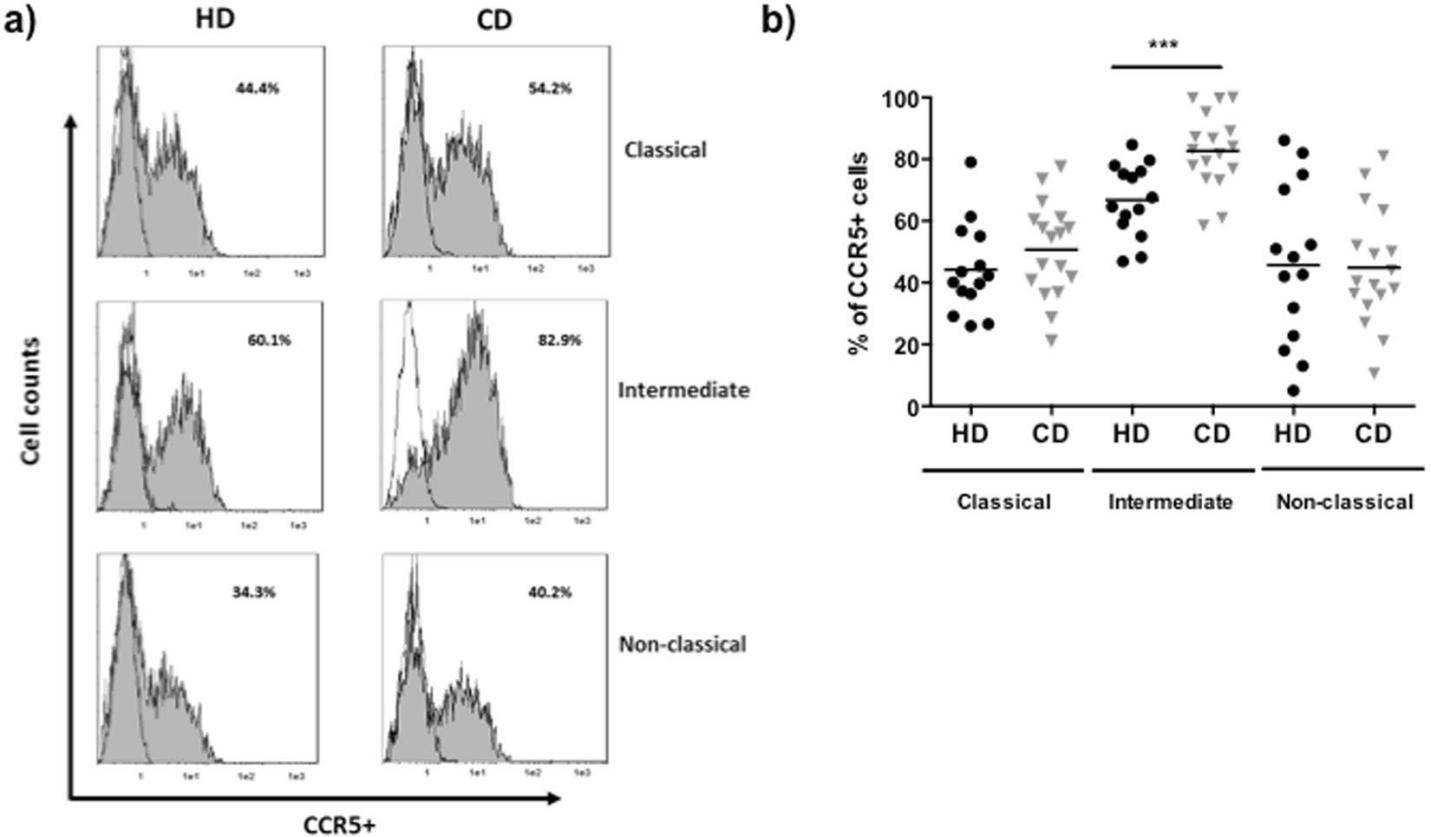
CCR5 expression on monocytes from HD and CD patients in remission. (**a**) A representative experiment of CCR5 expression on gated CD14+ CD16− (classical), CD14+ CD16+ (intermediate) and CD14low CD16++ (non-classical) monocytes from a HD and a CD patient by flow cytometry (unshaded histogram: isotype control, gray shaded histogram: anti-CCR5 antibodies). (**b**) The percentage of CCR5+ classical, intermediate and non-classical monocytes by flow cytometry at 24 h of WB culture from HD (n = 14) and inactive CD patients (n = 17). Multiple comparisons were calculated by ANOVA for HD (p = 0.004) and for CD patients (p < 0.0001). Mann-Whitney U test was used for comparisons between each subset of monocytes from HD and inactive CD patients (***p < 0.001).

We next assessed the expression of CCR5 on the different monocyte subsets after 24 h of WB culture. The percentage of classical, intermediate and non-classical monocytes were not different in CD and HD (classical monocytes HD: 28.69 ± 4.77%, CD: 31.87 ± 4.76%; intermediate monocytes HD: 58.46 ± 4.47%, CD: 55.75 ± 3.99%; non-classical monocytes: HD 2.57 ± 1.10%, CD: 1.62 ± 0.28%). There were no differences in the percentages of CCR5+ classical and non-classical monocytes from HD and CD (classical CCR5+ monocytes HD: 44.20 ± 3.92, CD: 50.71 ± 3.79; non-classical CCR5+ monocytes: HD: 45.72 ± 6.92, CD: 44.88 ± 4.54). However, the percentage of CCR5+ intermediate monocytes was higher in CD patients than in HD (intermediate CCR5+: HD 66.77 ± 3.14, CD: 82.73 ± 2.97, p < 0.001) (Fig. 1a,b).

### CSF-1 and IL-10 upregulates CCR5 expression

**Figure 2:**
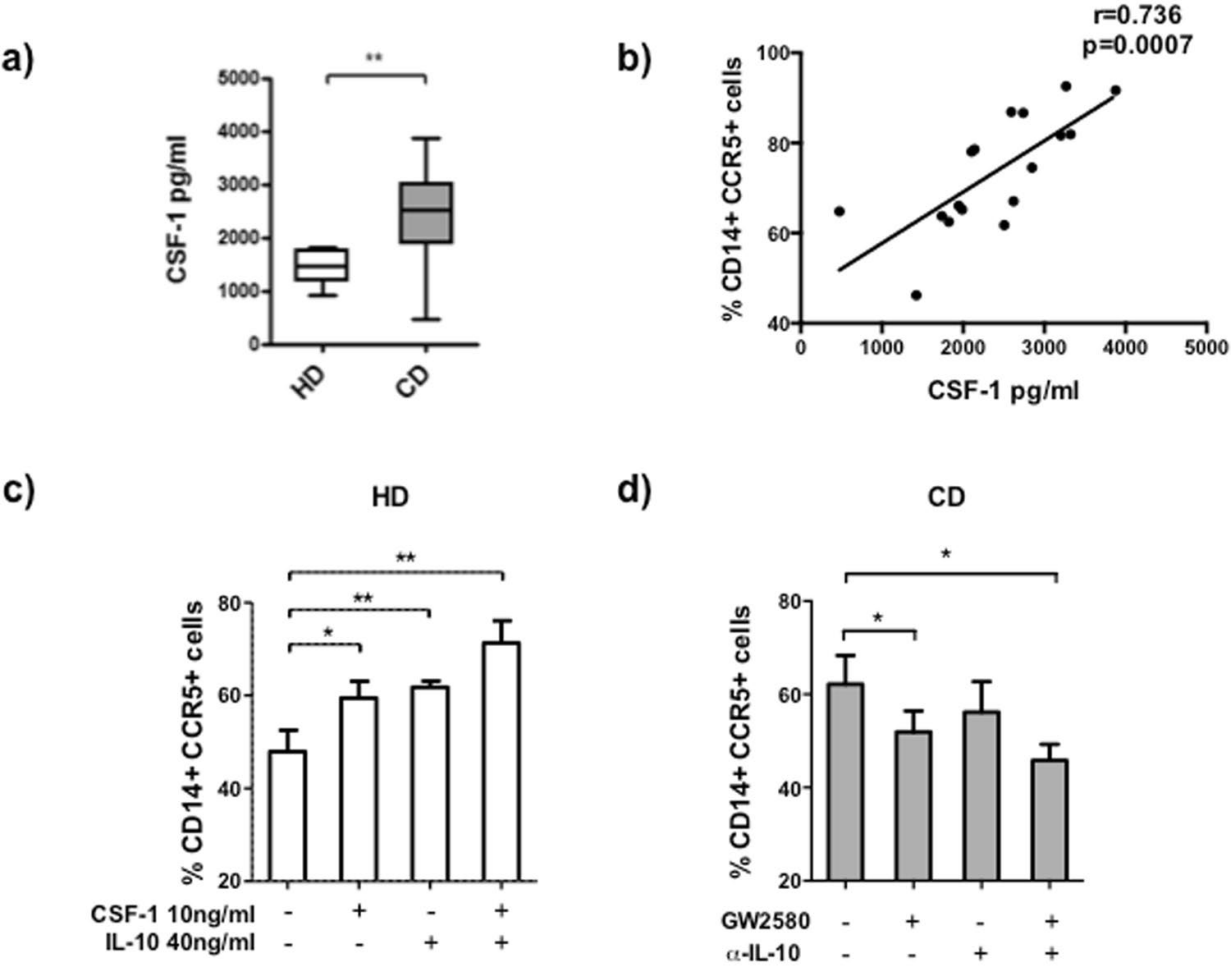
CSF-1 and IL-10 upregulated CCR5 expression on monocytes from HD and CD patients in remission. (**a**) The levels of CSF-1 after 24 h of WB culture of peripheral blood monocytes from HD (n = 14) and CD patients in remission (n = 17) were quantified by ELISA. (**b**) Correlation between CSF-1 levels and the percentage of CD14+CCR5+ peripheral blood monocytes from CD patients after 24 h of WB culture. (**c**) Peripheral blood monocytes from HD after 24 h of culture with medium, CSF-1 (10 ng/ml), IL-10 (40 ng/ml), or CSF-1+IL-10 were analyzed by flow cytometry. The results are expressed as a percentage of CD14+CCR5+ cells. (**d**) Peripheral blood monocytes from CD patients after 24 h of culture in the presence of medium, GW2580 (35 ng/ml), anti-IL-10 (3.5 ng/ml), or GW2580+anti-IL-10 were analyzed by flow cytometry. The results are expressed as a percentage of CD14+CCR5+ cells. Mann-Whitney U test was used for the comparison of CSF-1 concentrations between HD and CD patients. Spearman test was used to calculate the correlation between CSF-1 levels and CD14+CCR5+ cells. ANOVA was used for multiple comparisons of migration in HD (p < 0.0001) and CD (p = 0.05). Wilcoxon test was used for the comparisons between medium and each different condition in HD and CD. (*p < 0.05, **p < 0.01, ***p < 0.001).

To elucidate factors that could be responsible for the CCR5 upregulation in monocytes from CD patients, we measured the concentration of spontaneously produced cytokines in the supernatants of 24h-WB cultures. TNF-α, IL-10, and IL-6 concentrations in the supernatants from HD and CD patients were comparable (data not shown). The CSF-1 concentration was significantly higher in CD than in HD supernatants (HD: 1421 ± 120.9 pg/ml, CD: 2390 ± 199.80 pg/ml, p < 0.01) (Fig. 2a). The production of CSF-1 correlated positively with the percentage of CCR5+ monocytes (r = 0.736, p = 0.0007) (Fig. 2b) and with the percentage of CCR5+ intermediate monocytes (r = 0.695, p = 0.02). NOD2 polymorphism did have neither influence on the percentage of CCR5+ monocytes nor on the CSF-1 production (Supplementary Fig. S2). To determinate the influence of cytokines on the higher percentage of CCR5+ cells, HD monocytes were stimulated with IL-10, TNF-α, IL-6, and CSF-1. TNF-α and IL-6 did not upregulate the percentage of CCR5+ cells (data not shown). However, IL-10 or CSF-1 increased the percentage of CCR5+ cells without reaching CD levels. The stimulation of HD monocytes with both IL-10 and CSF-1 increased the percentage of CCR5+ cells to levels of CD monocytes (medium: 47.95 ± 4.60; CSF-1: 10 ng/ml: 59.47 ± 3.57, p < 0.05; IL-10: 40 ng/ml: 61.76 ± 1.34, p < 0.01; CSF-1 10 ng/ml+IL-10 40 ng/ml: 71.30 ± 4.80, p < 0.01) (Fig. 2c). Inversely, when we blocked IL-10 and CSF-1 in the WB culture of CD cells with specific anti-IL-10 or CSF-1 inhibitor (GW2580), the percentage of CCR5+ monocytes diminished without reaching HD levels. Only after blocking the CD WB-culture with both anti-IL-10 and GW2580, the percentage of CCR5+ monocytes was comparable to HD (Medium: 62.20 ± 6.14; GW2580 51.93 ± 4.49, p < 0.05; anti-IL-10: 56.16 ± 6.56; anti-IL-10+GW2580: 45.83 ± 3.45, p < 0.05) (Fig. 2d).

### Effects of IL-10 and CSF-1 on monocytes from CD patients and HD

**Figure 3:**
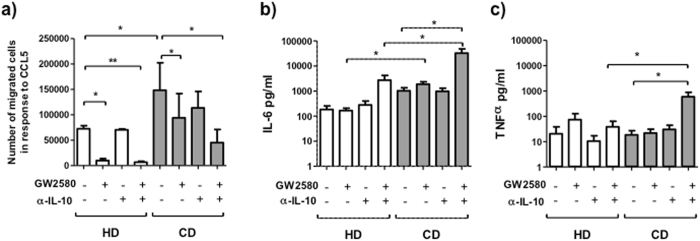
Functional effects of CSF-1 and IL-10 on peripheral blood monocytes from HD and CD patients in remission. (**a**) Peripheral blood monocytes from HD (n = 4) and inactive CD patients (n = 6) after 24 h of culture with medium, GW2580 (35 ng/ml), anti-IL-10 (3.5 ng/ml), or GW2580+anti-IL-10 were subjected to a 4h-chemotaxis assay toward CCL5. The cells that had migrated into the lower chamber were collected and stained with anti-CD14 and analyzed by flow cytometry. The results are expressed as the mean of the number of CD14+ migrated cells. (**b**,**c**) The levels of IL-6 and TNF-α in the supernatant of the 24h-culture of peripheral blood monocytes from HD and inactive CD in the presence of medium, GW2580 (35 ng/ml), anti-IL-10 (3.5 ng/ml), or GW2580+anti-IL-10 were quantified by ELISA. Multiple comparisons of HD and CD were calculated by ANOVA (migrated cells HD: p < 0.0001 and CD: p = 0.05; IL-6 concentration in HD: p = 0.10 and CD: p = 0.09; TNF-α concentration in HD: p = 0.30 and CD: p = 0.05). The Mann-Whitney U test was used for comparison of HD and CD in each culture condition, and Wilcoxon test was used for the comparison between medium and each culture in HD and in CD (*p < 0.05, **p < 0.01).

To determine the effects of IL-10 and CSF-1 on the migration of CD and HD monocytes, a chemotaxis assay with CCL5 was performed with WB-cultured monocytes in the presence or absence of blocking anti-IL-10 antibodies or GW2580. After 24 h in medium, the number of CD monocytes that migrated toward CCL5 was significantly higher than the number of HD monocytes (HD: 72640 ± 5928; CD: 148491 ± 4008; p < 0.05). After 24 h in medium with blocking anti-IL-10 antibodies and GW2580, the numbers of migrated CD and HD monocytes were significantly lower than in medium (HD: 6757 ± 1822; CD: 45233 ± 25918; p < 0.01 and p < 0.05 respectively) (Fig. 3a). More intermediate monocytes of CD patients than of HD migrated toward CCL5 (intermediate: HD: 85033 ± 4921, CD: 174933 ± 59646; p < 0.05). Blocking with IL-10 and GW2580 reduced significantly the migration of these monocytes (Supplementary Fig. S3).

CD monocytes produced higher levels of TNF-α and IL-6 than HD monocytes after 24 h of culture with both anti-IL-10 antibodies and GW2580 (IL-6: HD: 3222 ± 1927, CD: 32595 ± 15615, p < 0.05; TNF-α: HD: 38.40 ± 25.54, CD: 591 ± 290.2, p < 0.05) (Fig. 3b,c).

### CCL5/CCR5 signaling modifies phenotype during the maturation of monocytes from HD and CD patients

**Figure 4:**
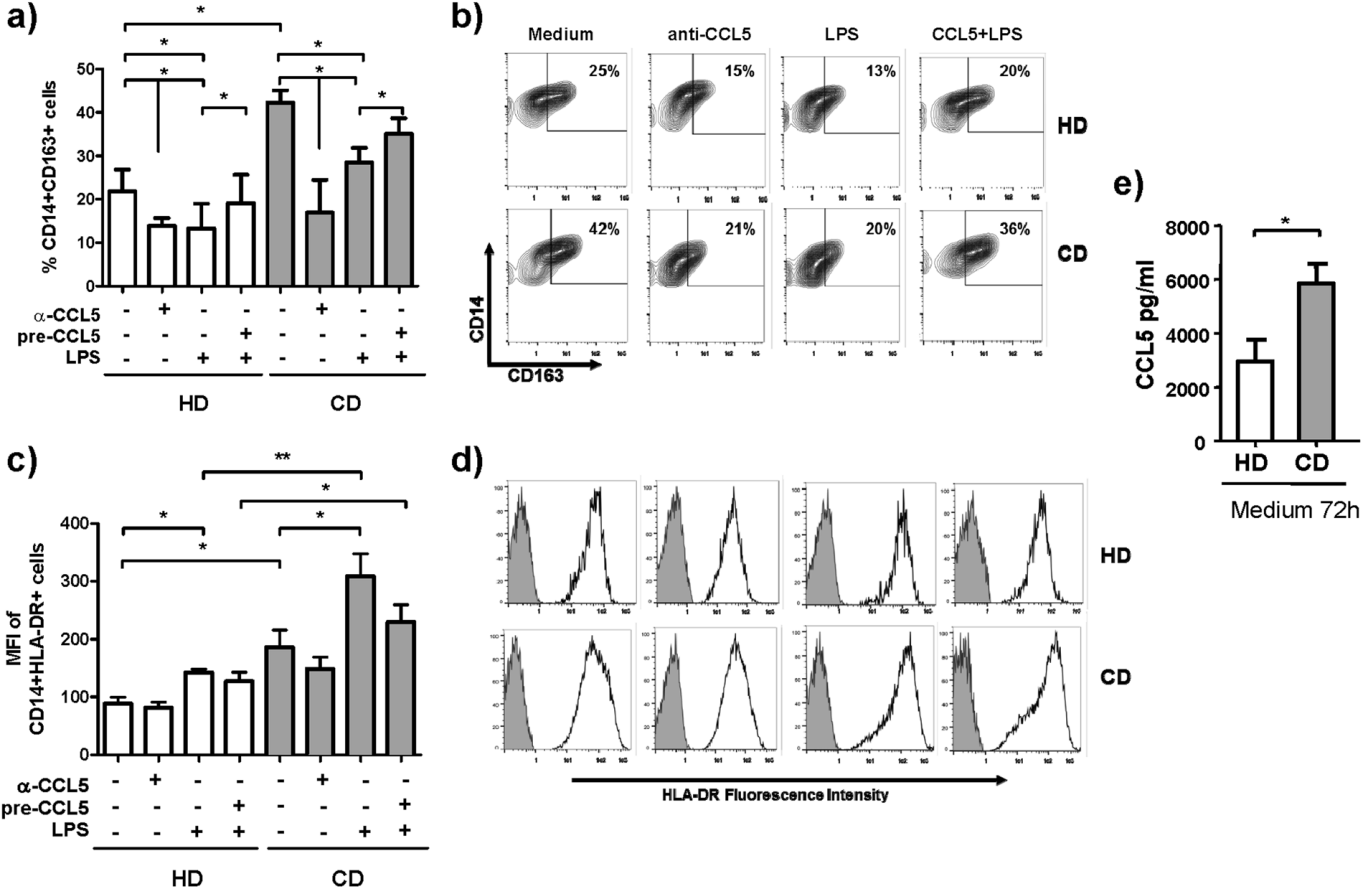
CCL5/CCR5 signaling modified the phenotype of monocytes during their maturation. (**a**,**c**) Peripheral blood monocytes from HD (n = 6) and CD patients (n = 7) were cultured with medium or with anti-CCL5 (10 ug/ml) for 72 h. Other peripheral blood monocytes from the same donors were cultured in medium for 24 h, then washed and stimulated for 24 h with or without CCL5 (10 ng/ml) (pre-CCL5). These cells were then washed and stimulated for 24 h with LPS (10 ng/ml). At the end of the cultures, cells were washed and stained with anti-CD14, anti-CD163, and anti-HLA-DR and were analyzed by flow cytometry. Results are expressed as the percentage of CD14+CD163+ cells and the mean fluorescence intensity (MFI) of CD14+HLA-DR+ cells. (**b**,**d**) A representative experiment of the CD163 and HLA-DR expression on peripheral blood monocytes after 72 h of culture with each described condition. Dot plot shows the percentage of CD14+ and CD163+ from HD and inactive CD patients. Histogram shows the expression of HLA-DR on gated CD14+ monocytes from HD and CD patients (gray shaded histogram: isotype control, unshaded histogram: anti-HLA-DR antibodies). (**e**) CCL5 concentration in the supernatants of peripheral blood monocytes from HD and CD patients after 72 h in medium was quantified by ELISA. Multiple comparisons were calculated by ANOVA (% of CD163+ monocytes in HD: p = 0.19 and in CD: p = 0.01; MFI of HLA-DR in HD: p = 0.40 and in CD: p = 0.09). Mann-Whitney U test was used for comparisons between HD and CD patients. Wilcoxon test was used for the comparisons between medium and each condition in HD and CD patients (*p < 0.05, **p < 0.01).

We compared the phenotypic changes of CD and HD monocytes that were pre-cultured with or without blocking anti-CCL5 antibodies. After 72 h of stimulation, monocytes were stained with antibodies specific for HLA-DR and scavenger receptors (CD163 and CD206). Monocytes were then gated based on CD14+ expression. In the culture without anti-CCL5 antibodies, the percentages of CD206+ cells were similar in CD and HD monocytes (data not shown). However, HLA-DR levels and the percentage of CD163+ monocytes were significantly higher in CD patients than in HD (HLA-DR MFI on monocytes with medium: HD: 88.52 ± 11.23, CD: 185.9 ± 29.71, p < 0.05; CD163+ cells with medium: HD 25.19 ± 6.97% and CD 42.79 ± 5.52% p < 0.05) (Fig. 4). In the culture with anti-CCL5 antibodies, HLA-DR expression on CD and HD monocytes was not different. With anti-CCL5 antibodies, the percentage of CD163+ CD and HD monocytes was lower than with medium (HD: 13.86 ± 1.85, CD: 16.92 ± 7.50; medium vs. anti-CCL5 p < 0.05) (Fig. 4). These findings suggest that CCL5 was responsible for the CD163 upregulation on CD monocytes.

To confirm the influence of CCL5 on the response to inflammatory stimuli, CD and HD monocytes were cultured 24 h with or without recombinant CCL5 (10 ng/ml) before the stimulation with LPS (10 ng/ml). Cells were then collected and stained with anti-CD14, anti-CD16, anti-CD206, anti-HLA-DR, and anti-CD163 antibodies and analyzed by flow cytometry. The percentages of CD206+ and CD16+ monocytes in CD were similar to HD. HLA-DR expression in CD monocytes stimulated with LPS was significantly higher than in HD monocytes (HD: 142.40 ± 5.84, CD 308.50 ± 39.21, p < 0.01). There were no significant HLA-DR differences in monocytes pre-treated with or without CCL5 (pre-CCL5+ LPS: HD: 127.10 ± 15.76, CD: 229.31 ± 29.95) (Fig. 4c,d). The percentage of CD163+ monocytes was lower if they were stimulated with LPS than if they were in medium. The CCL5 pre-treatment abrogated the LPS-induced downregulation of CD163+ CD and HD monocytes (HD: 13.78 ± 5.94, CD: 28.14 ± 5.45; pre-CCL5+ LPS: HD: 19.49 ± 5.96, CD: 35.07 ± 4.25; p < 0.05) (Fig. 4a,b). We next compared the intrinsic CCL5 production by HD and CD monocytes that could explain the observed phenotype differences on HD and CD monocytes. In the 72 h-supernatants, the CCL5 concentration was higher in the culture from CD monocytes than in the culture from HD monocytes (HD: 2966 ± 791.7 pg/ml, CD: 5846 ± 739.5 pg/ml; p < 0.05) (Fig. 4e).

### CCL5 suppressed LPS-induced IL-6 and TNF-α production by CD monocytes

**Figure 5:**
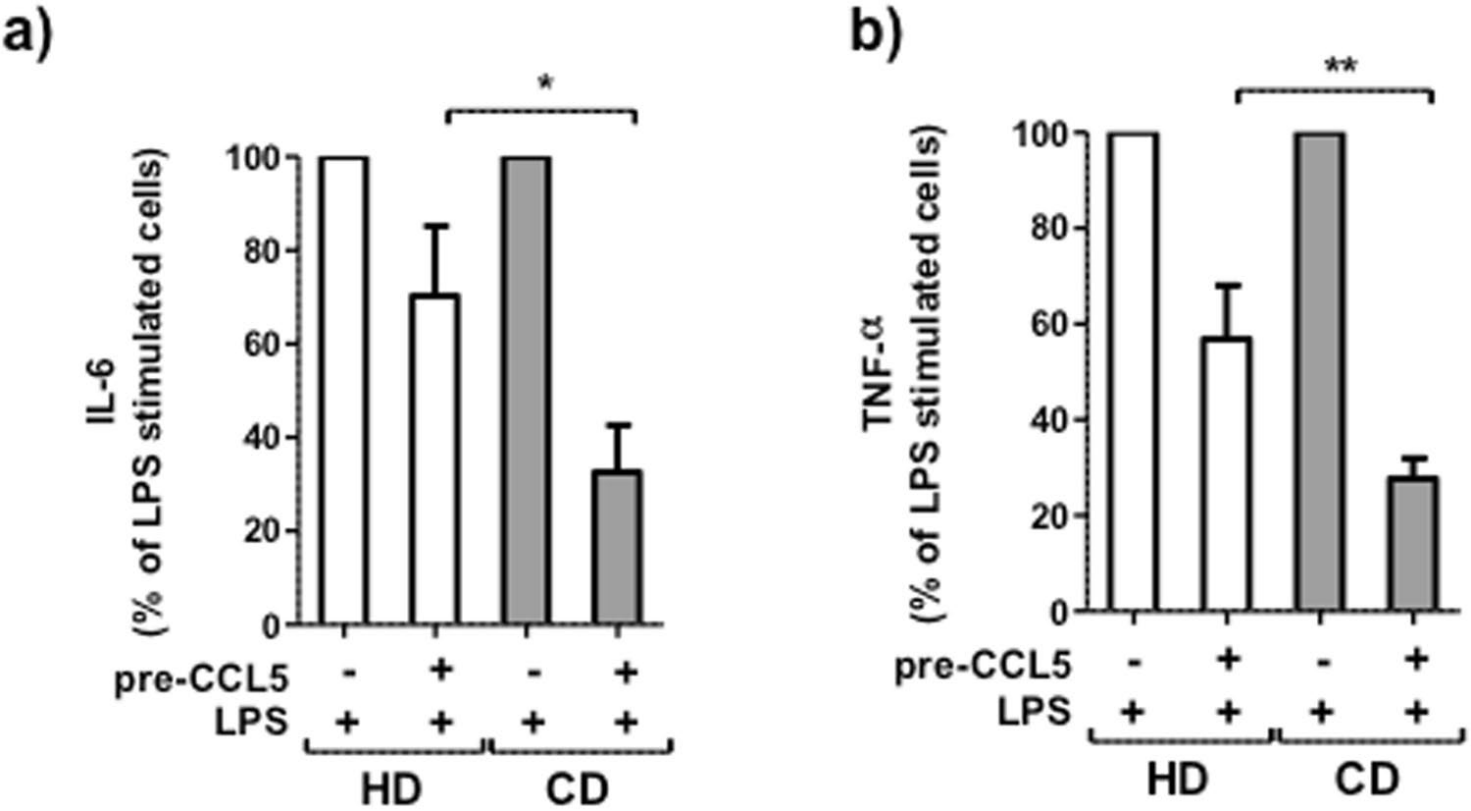
CCL5 suppressed LPS-induced IL-6 and TNF-α production by peripheral blood monocytes from HD and CD patients in remission. (**a**,**b**) Peripheral blood monocytes from HD (n = 6) and CD patients (n = 7) were cultured in medium alone for 24 h; then, cells were washed and stimulated with or without CCL5 (pre-CCL5) for 24 h. Subsequently, cells were washed and stimulated with or without LPS. The levels of IL-6 and TNF-α in the supernatants were quantified by ELISA. Mann-Whitney U test was used for the comparisons between HD and CD patients (*p < 0.05, **p < 0.01).

To evaluate the effect of CCL5 on cytokine production, monocytes from CD patients and HD were pre-treated with or without CCL5 prior to LPS stimulation. Pre-treatment with CCL5 suppressed LPS-induced IL-6 and TNF-α production by HD and CD monocytes. The suppression was higher in the cultures with monocytes from CD patients than with monocytes from HD (IL-6: HD: 30%, CD: 70% p < 0.05: TNF-α: HD: 40%, CD: 75% p < 0.01) (Fig. 5).

### CCR5 expression and CSF-1 were associated with clinical behavior and treatment in CD patients in remission

**Figure 6:**
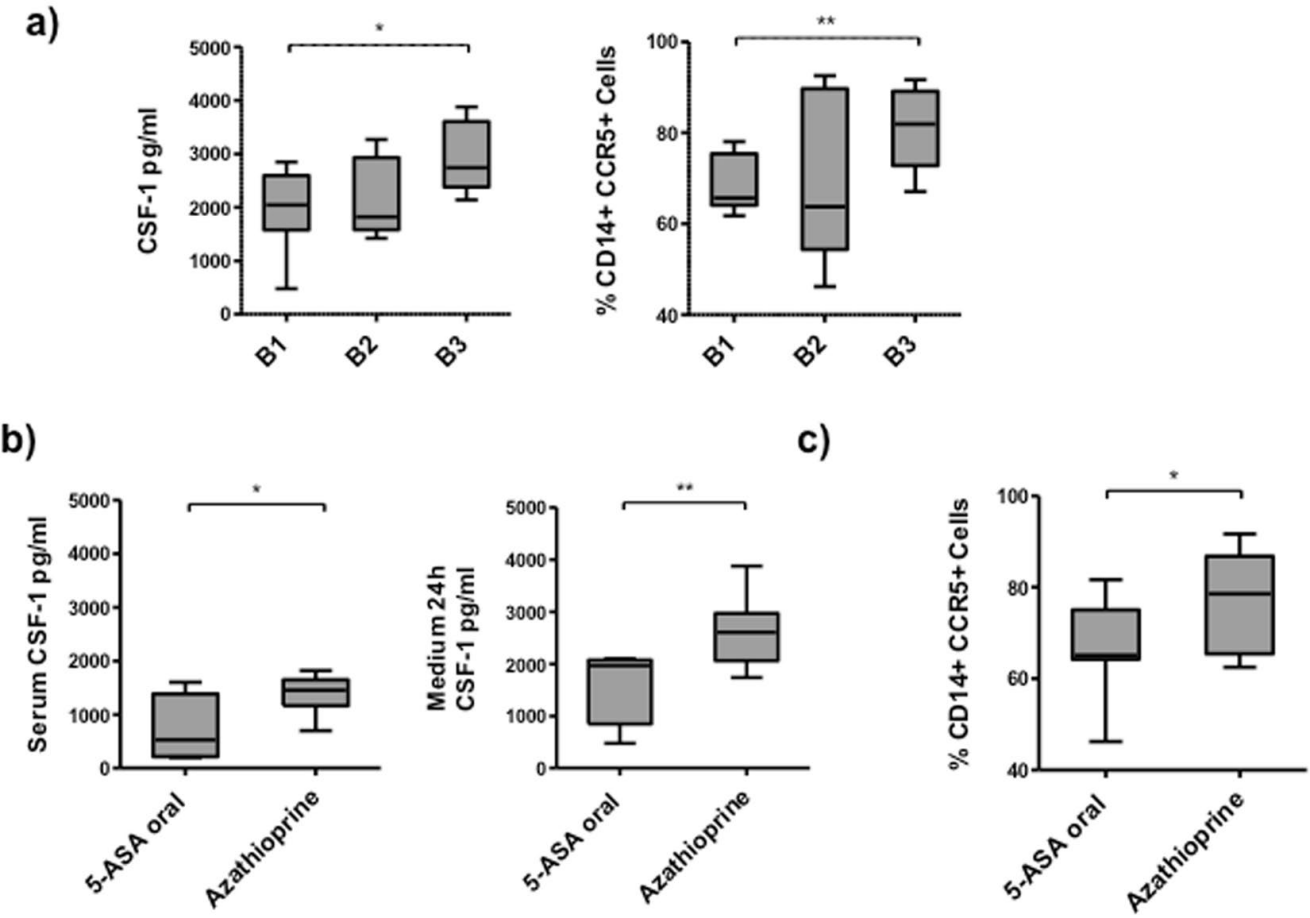
CSF-1 and CCR5 expression was associated with clinical behavior and treatment in CD patients in remission. (**a**) CSF-1 concentration and the percentage of CD14+CCR5+ monocytes after 24 h of culture of peripheral blood from CD patients (n = 17) segregated according to clinical behavior (B1, B2, and B3). Multiple comparisons were calculated by ANOVA for levels of CSF-1 and the percentage of CCR5+ monocytes in regard to clinical behavior (p = 0.16, p = 0.08). (**b**) Concentration of CSF-1 in serum and in the supernatant of 24h-cultured peripheral blood monocytes from CD patients segregated according to treatment (5-ASA and azathioprine). (**c**) Percentage of CD14+CCR5 monocytes from CD patients after 24 h of culture according to treatment (5-ASA and azathioprine). Wilcoxon test was used for the comparisons between treatment groups (*p < 0.05, **p < 0.01).

We next analyzed the relationship between CCR5 expression, CSF-1 production, and clinical parameters in our cohort of CD patients (Montreal classification: B1 (without stricture formation; non-penetrating), B2 (with stricture formation), and B3 (internally penetrating); location, treatment, age of diagnosis, smoke, and previous surgery). When we segregated patients according to the disease behavior (Montreal classification), B3 patients showed the highest levels of CSF-1 in 24h-culture supernatants and the highest CCR5 expression (CSF-1 pg/ml: B1: 1978 ± 332.10, B2: 2170 ± 335.30, B3: 2940 ± 300.71; B1 vs. B3: p < 0.05; percentage of CCR5+ cells: B1: 68.43 ± 2.60, B2: 70.40 ± 8.52, B3: 81.19 ± 4.15; B1 vs. B3: p < 0.01) (Fig. 6a). Patients treated with azathioprine showed higher levels of CSF-1 in serum and in 24h-culture supernatants, and higher CCR5 expression than patients treated with 5-ASA (5-ASA: 730 ± 50 pg/ml, azathioprine: 1388.60 ± 156.27 pg/ml, p < 0.05, 24 h medium pg/ml: 5-ASA: 1628 ± 385.50, azathioprine: 2621 ± 205.40, p < 0.05; percentage of CCR5+ cells: 5-ASA: 66.39 ± 3.75, azathioprine: 77.08 ± 3.57, p < 0.05) (Fig. 6b,c).

## Discussion

Our findings showed a higher CCR5 upregulation on CD monocytes than on HD monocytes induced by a higher CSF-1 and IL-10 production in CD than in HD cultures. With this upregulation, the migratory pattern and the maturation status in CD monocytes were different from HD monocytes. This CSF-1 and CCR5 upregulation in CD patients was related to penetrating disease, azathioprine treatment, and the lowest levels of systemic inflammation.

The expression of CCR5 on the surface of freshly isolated monocytes from HD and CD patients in remission was lower by flow cytometry in our experimental conditions. However, we observed, in concordance with previous reports, that after culturing in medium, 45% of HD monocytes and more than 70% of CD monocytes expressed CCR5^22, 23^. This 20 h-culture could have initiated monocyte differentiation because the induction of CCR5 expression is characteristic of the transition from monocytes to macrophages^23–25^. In addition, our findings suggest that specific soluble factors produced during the 24 h WB cell culture could have induced a higher expression of CCR5 in CD monocytes and initiated a faster differentiation to macrophages.

Several authors have reported a higher level of expression of CCR5 in monocytes from patients with acute liver failure and rheumatoid arthritis^26, 27^. In rheumatoid arthritis (RA), the circulating CD14+ CD16+ monocytes expressed higher CCR5 levels than classical monocytes^28, 29^. This phenotype suggests that blood monocytes from RA patients turn into tissue-infiltrative CD16+ cells before entry into the joint. These CD14+ CD16+ monocytes from RA patients also express CD16, CD163, and lower levels of HLA-DR and produce lower levels of TNF-α, IL-1β, and IL-6 but higher levels of IL-10 than the other monocytes^19, 28, 29^. This is a similar phenotype to the monocytes cultured in the presence of CCL5.

Since CCR5/CCL5 modulates the inflammatory response induced by TLR4 ligands^9^, the observed higher expression of CCR5 on CD monocytes could have functional consequences. Accordingly, we showed that the blockage of both CCR5 and IL-10 signals reduced the migratory potential and the percentage of CD14+CD163+ monocytes, but increased the TNF-α and IL-6 release induced by TLR4 ligands. Conversely, when we added CCL5, the percentage of CD14+CD163+ monocytes increased and the TNF-α and IL-6 production and HLA-DR expression were attenuated. Shahrara *et al.* showed that CCL5 decreases LPS-induced IL-6 transcriptionally and TNF-α post-transcriptionally^9^. This CCL5-induced modulation of TLR4-induced TNF-α and IL-6 secretion suggests the non-inflammatory polarization of monocytes in the presence of CCL5. We analyzed the signaling through CCR5 and its relationship with monocyte activation. Our findings are concordant with a report describing that CCR5/CCL5 signaling increased the gene expression of CD163 on monocytes^30^. CD163 is known as M2-like macrophage marker^31^. Thus, monocyte maturation into tissue macrophages in the presence of CSF-1, accompanied by an increased expression of CCR5, could be a maturation process towards M2-like CD163+ macrophages. On the surface of CD monocytes, higher CCR5 expression may represent a higher sensitivity to CCL5 and may facilitate the transition to become resident or M2-like CD163+ macrophages. A similar conclusion was obtained with studies using synovial fluids from patients with RA, where the neutralization of CCL5 increased the TNF-α levels and reduced the IL-10 levels induced by LPS^9^.

In addition to the higher CCR5 upregulation on CD monocytes, we showed that these cells produced higher CCL5 levels than HD monocytes. This CCL5 increase in CD patients is not exclusively due to the production by circulating monocytes because several reports have also showed a higher intestinal secretion of CCL5^14, 17, 20, 27^. In a CD context with higher CCR5 and CCL5, the cross-linking of CCR5 will induce the synthesis of high levels of CSF-1, CCL5, MIP-1α, and MIP-1β and will contribute to enhancing the survival of macrophages^32^.

In addition to the CCR5 upregulation, we showed that monocytes from CD patients in remission produced higher amounts of CSF-1. CSF-1 expression and serum CSF-1 have been shown to be increased in IBD patients and in experimental colitis^33^. Zwicker *et al*. showed that monocytes from IBD patients had a tendency to higher mRNA CSF-1 expression^34^. CSF-1 is a key regulator of the differentiation, proliferation, and survival of monocytes. Macrophages that are differentiated *in vitro* from human CD14+ monocytes under the influence of CSF-1 polarized toward an anti-inflammatory phenotype^35^. The expression of TNF-α and IL-1β is thus decreased in CSF-1-differentiated macrophages. In mucosa, CSF-1 has additional protective functions by recruiting tissue macrophages that promote cell proliferation and the survival of epithelial cells^36–38^. Therefore, CSF-1 rich conditions seem to contribute to the improvement of pathological conditions in patients with IBD. Certain therapies provide clinical efficacy through the increase in CSF-1^39^.

CSF-1 is not always associated with a protective function. Several researchers have found higher levels of CSF-1 in serum of patients with different pathologies and their relation with prognosis^40–42^. Patients with chronic coronary artery disease with CRP levels >2.5 mg/L had a 13-fold risk of future events when their CSF-1 levels were higher than when they were lower^43^. In fact, it has been noted that higher CRP levels induce CSF-1 production and proliferation of macrophages^44, 45^.

Our findings suggest that the increased CSF-1 levels are responsible for the CCR5 upregulation in monocytes from CD patients. In HD monocytes, the addition of CSF-1 markedly increased the surface expression of CCR5^46^. It has been shown that CSF-1 causes the greatest upregulation of CCR5 in macrophage maturation compared with other growth factors and cytokines^47^. In rheumatoid arthritis, CCR5 increased expression has also been related with higher levels of CSF-1 and IL-10^29^. The CSF-1-induced upregulation of CCR5 in differentiating monocytes has other consequences. In liver lesions, CSF-1 is profibrotic because it increases the infiltrating capacity of monocytes in a CCR5-dependent manner and drives the differentiation of monocytes to M2 macrophages^48^.

From our results, we can speculate that azathioprine acts through the CSF-1-CCR5-regulatory pathway on monocytes from CD patients in remission. We observed that CD patients treated with azathioprine had the highest levels of CSF-1 and CCR5 expression. Azathioprine is an effective immunomodulator and its mechanism of action is not limited to its cytostatic properties on lymphocytes. Its active form, 6-thioguanine, also seems to regulate monocytes. In this regard, Nazareth *et al*. showed that macrophages from CD patients treated with azathioprine secreted more IL-10 and less TNF-α than macrophages from untreated CD patients in response to bacterial infection^49^. In the present study, we associated the azathioprine treatment with higher CSF-1 levels and CCR5 expression in the monocytes.

When some authors described CCR5 KO mice as protected from DSS-induced colitis, this receptor became a potential therapeutic target in CD. However, our results, along with others showing the regulatory/anti-inflammatory role of the CSF-1-CCR5 pathway, suggest that targeting CCR5 has a detrimental proinflammatory effect^9^. It is therefore not surprising that the CCR5 targeting strategy has not been an efficacious treatment in CD patients. Further research is necessary to apply therapies targeting the migratory pattern through CCR5 in CD patients.

## Material and Methods

### Patients and Samples

Whole blood samples were collected from 31 HD that were age and sex matched with 21 CD patients who met the inclusion criteria. All patients had definitive diagnoses of CD and they were in remission, which was confirmed by standard diagnostic criteria (Harvey Bradshaw Index [HBI] <3) Patients receiving either a stable maintenance dose of 5-aminosalicylates (5-ASA) (2.5 g/d) or azathioprine (1.5 to 4 mg/kg day) for the previous 6 months were included. None of the patients had received conventional/systemic corticosteroids, anti-TNF-α, or metronidazole therapy within the previous 6 months. Only patients with no surgery during the last year were included (Table 1). Samples were processed within one hour after collection and maintained at room temperature. The WB was collected into Vacutainers (BDBiosciences, Franklin Lakes, NJ, USA) containing heparin sodium. Plasma from HD and CD patients was also recollected and kept at −80 °C until use.

**Table 1:** Clinical characteristics of the Crohn’s disease study population.

Characteristic	(*n* = *21*)
***Age*** **(** ***years*** **) (** ***mean*** **(** ***SD*** **))**	51.89 (13.38)
***Sex*** **(** ***n*** **) (** ***M/F*** **)**	(10/11)
***Years from diagnosis*** **(** ***mean*** **(** ***SD*** **))**	19.40 (9.25)
***Montreal L-category*** **(** ***n*** **)**	
L1/L2/L3	10/5/6
***Montreal B-category***	
B1/B2/B3	8/7/6
***Smoke***	7
***Therapy***	
None (n)	0
5-ASA (n)	10
Immunosuppressors (n)	11
Infliximab (n)	0
Antibiotics (n)	0
***Surgery*** **(** ***n*** **)**	9

### Ethical statement

The study included consecutive outpatients treated at the Hospital de la Santa Creu i Sant Pau, a tertiary care hospital in Barcelona, Spain. The protocol conformed to the Declaration of Helsinki and Guidelines for Good Clinical Practice in Clinical Trials and was approved by the Clinical Research Ethics Committee of the same hospital. All patients received information concerning their participation in the study and gave written informed consent.

### Reagents

Recombinant CSF-1, IL-10, and CCL5 were obtained from Peprotech (London, UK), GW2580 (anti- CSF-1) from Sigma-Aldrich (St. Louis, Missouri), anti-IL-10 from BDBiosciences, Lipopolisaccharide (LPS) from InvivoGen (tlrl-pelps, San Diego, CA, USA), and anti-CCL5 from Antibodies-online GmbH (Aachen, Germany). All concentrations were used such as it were described in previous works and tested previously in HD donors^9, 50–52^. Cells were stained with LIVE/DEAD fixable dead cell stain kit (Invitrogen, Carlsbad, CA, USA) to test viability after 24 h of culture with GW2580. Only samples with >95% of cellular viability were included.

### Whole blood cell culture

Whole blood cells (2.5 ml) were cultured in 1 ml of RPMI 1640 medium (Biowhittaker, Verviers, Belgium) or medium plus CSF-1 (10 ng/ml), IL-10 (40 ng/ml), anti-IL-10 (35 μg/ml) concentrations of CSF-1 and IL-10 were tested in HD or GW2580 (35 ng/ml), or. Cultures were maintained at 37 °C with 5%CO_2_ for 24 h. To determine the influence of CCL5/CCR5 signaling on the maturation of monocytes, WB cells were pre-cultured with CCL5 (10 ng/ml) (pre-CCL5), anti- CCL5 (5 μg/ml) (anti-CCL5), or medium. After 24 h, pre-cultured cells were washed and stimulated with LPS (10 ng/ml) or medium for 24 h^9^.

### Whole blood cell staining and flow cytometry analysis

The expression of chemokine receptors was determined on the surface of fresh monocytes or cultured monocytes^53, 54^. Cells were incubated for 20 min at room temperature in the dark with anti-CD14, anti-CD163 (BDBioscience) anti-CD16 (Immunotools, Friesoythe, Germany), anti-CCR2 (R&D Systems, Weisbaden, Germany), anti-CCR5 (Clone:HEK/1/85a), anti-CXCR4, anti-CX3CR1, anti-CD206, and anti-HLA-DR (Biolegend, San Diego, CA) monoclonal-antibodies. Red blood cells were then lysed using RBC lysis buffer (1X) (Biolegend). Cells were washed twice with staining buffer (PBS, supplemented with 0.5% BSA; Calbiochem Merck, Darmstadt, Germany) and resuspended in 200 μl of staining buffer to be analyzed by flow cytometry.

The surface expression of different markers was analyzed on gated CD14+ monocytes and on gated monocyte subsets (identified as CD14+ CD16- (classical), CD14+ CD16+ (intermediate) and CD14lowCD16++ (non-classical)) using a MACSQuant® Instrument (Miltenyi Biotec GmbH, Bergisch Gladbach, Germany). The percentage of positive cells (% cells) and mean fluorescence intensity (MFI) of each individual marker were calculated using © FlowJo, LLC 2013-2016 data analysis software.

### Determination of cytokine plasma levels

CSF-1, CCL5 (PeproTech, London, UK), TNF-α, IL-10 (BDBioscience), and IL-6 (Immunotools) levels were tested using the respective specific ELISA kit, according to the manufacturer’s instructions. All cytokines were quantified with the provided standard curves. The limits of detection were: 40 pg/ml for CSF-1, 16 pg/ml for CCL5, 10 pg/ml for IL-10, 20 pg/ml for TNF-α, and 8 pg/ml for IL-6.

### Chemotaxis assay

The chemotaxis of WB monocytes in response to recombinant human CCL5 (Peprotech) was measured across 3 µm pore-size cell culture inserts in 24-well companion plates (Millicell cell culture inserts) (Millipore, Billerica, MA). Medium supplemented (or not) with recombinant human CCL5 (100 ng/ml) was placed in the lower chamber of the plate. WB cultures were lysed with RBC lysis buffer (1X) (Biolegend), and cells were resuspended and adjusted at 3 × 10^5^ cells/ml in 150 μl of culture medium and placed into culture inserts. Plates were then incubated for 4 h at 37 °C (5% CO_2_) and those cells that had crossed the membrane were collected and incubated for 20 min at room temperature with 10 µl of anti-CD14 PECy7 mAbs (BDBiosciences) and anti-CD16 FITC (Immunotools). Cells were washed, counted, and analyzed by flow cytometry.

### Statistics

Statistical analyses were performed using the probe’s ANOVA, t-test, Mann-Whitney, Wilcoxon, and Fisher exact test on Graph Pad Prism 5 software. The nonparametric correlation Spearman test was applied to the analysis. Data were presented as mean ± SEM deviation. P-values < 0.05 were considered significant.

## Electronic supplementary material

Supplementary figures
