# Supplementary material for: CSF-1 regulates the function of monocytes in Crohn’s disease patients in remission
Source: Sci Rep. 2017 Mar 7;7:92. doi: 10.1038/s41598-017-00145-4 (PMC5427917; doi:10.1038/s41598-017-00145-4)
Supplement: Supplementary file 1 — Supplementary figures [file 41598_2017_145_MOESM1_ESM.docx]

**CSF-1 regulates the function of monocytes in**

**Crohn´s disease patients in remission**

Juan Camilo Nieto^1^*, Carlos Zamora^1^, Elisabet Cantó^1^, Esther Garcia-Planella^3^, Jordi Gordillo^3^, Maria Angels Ortiz^1^, Cándido Juárez^1,2^, Silvia Vidal^1^*

^1^Institut d’Investigacions Biomèiques (IIB) Sant Pau, Barcelona, Spain

^2^ Department of Immunology, Hospital de la Santa Creu i Sant Pau, Barcelona, Spain

^3^ Department of Gastroenterology, Hospital de la Santa Creu i Sant Pau, Barcelona, Spain

***CORRESPONDING AUTHORS**

Juan Camilo Nieto, PhD

Institut d’Investigacions Biomèdiques (IIB) Sant Pau.

Sant Antoni Maria Claret 167, 08025 Barcelona- Phone (34) 93-5538065

E-mail: [jcnietos2@gmail.com](mailto:jcnietos2@gmail.com)

Silvia Vidal, PhD

Institut d’Investigacions Biomèdiques (IIB) Sant Pau

Sant Antoni Maria Claret 167, 08025 Barcelona-Phone (34) 93-5537544

E-mail: [svidal@santpau.cat](mailto:svidal@santpau.cat)

**SUPPLEMENTARY FIGURES**

**Supplementary Fig. S1 Chemokine receptor expression in monocytes from HDs and CD patients in remission.** a) Representative experiment of chemokine receptors’ expression on peripheral blood monocytes from an HD and an inactive CD patient. Peripheral blood monocytes were stained at the time to extraction (T=0) and after 24h of WB culture with anti-CD14 antibodies and anti-CCR2, -CCR5, -CXCR4, and -CX3CR1 antibodies. Expression was assessed by flow cytometry analysis on gated CD14+ cells (Dotted line: Isotype control, unshaded histogram: HDs and gray shaded: CD). b) The percentage of CD14+ CCR5+ peripheral blood monocytes by flow cytometry at 24h of WB culture from HDs (n=31) and inactive CD patients (n=21). Mann Whitney test was used for comparisons between HDs and inactive CD patients (*** p<0.001).

**Supplementary Fig. S2. CSF-1 production and CCR5 expression on monocytes from CD patients carrying a NOD2 mutation or wild-type (WT) CD patients.** Concentration of CSF1 and percentage of CD14+CCR5+ monocytes.

**Supplementary Fig. S3.** **Effects of CSF-1 and IL-10 on the migration of monocyte subsets**. Classical, intermediate and non-classical monocytes from HDs and CD patients were cultured with medium, GW2580 (35ng/ml), anti-IL-10 (3.5ng/ml), or GW2580+anti-IL-10. After 24h, cells were subjected to a 4h-chemotaxis assay toward CCL5. The cells that had migrated into the lower chamber were collected and stained with anti-CD14 and anti-CD16 and were analyzed by flow cytometry. The results are expressed as the mean of the number of migrated cells. Multiple comparisons were calculated by ANOVA (HDs p= 0.17 and CD p=0.04).
